# Message Frame–Tailoring in Digital Health Communication: Intervention Redesign and Usability Testing

**DOI:** 10.2196/33886

**Published:** 2022-04-21

**Authors:** Inge S van Strien-Knippenberg, Maria B Altendorf, Ciska Hoving, Julia C M van Weert, Eline S Smit

**Affiliations:** 1 Department of Communication Science Amsterdam School of Communication Research University of Amsterdam Amsterdam Netherlands; 2 Department of Health Promotion School for Public Health and Primary Care Maastricht University Maastricht Netherlands

**Keywords:** digital health communication, web-based computer tailoring, smoking cessation intervention, message framing, usability testing

## Abstract

**Background:**

Message frame–tailoring based on the need for autonomy is a promising strategy to improve the effectiveness of digital health communication interventions. An example of a digital health communication intervention is Personal Advice in Stopping smoking (PAS), a web-based content-tailored smoking cessation program. PAS was effective in improving cessation success rates, but its effect sizes were small and disappeared after 6 months. Therefore, investigating whether message frame–tailoring based on the individual’s need for autonomy might improve effect rates is worthwhile. However, to our knowledge, this has not been studied previously.

**Objective:**

To investigate whether adding message frame–tailoring based on the need for autonomy increases the effectiveness of content-tailored interventions, the PAS program was redesigned to incorporate message frame–tailoring also. This paper described the process of redesigning the PAS program to include message frame–tailoring, providing smokers with autonomy-supportive or controlling message frames—depending on their individual need for autonomy. Therefore, we aimed to extend framing theory, tailoring theory, and self-determination theory.

**Methods:**

Extension of the framing theory, tailoring theory, and self-determination theory by redesigning the PAS program to include message frame–tailoring was conducted in close collaboration with scientific and nonscientific smoking cessation experts (n=10), smokers (n=816), and communication science students (n=19). Various methods were used to redesign the PAS program to include message frame–tailoring with optimal usability: usability testing, think-aloud methodology, heuristic evaluations, and a web-based experiment.

**Results:**

The most autonomy-supportive and controlling message frames were identified, the cutoff point for the need for autonomy to distinguish between people with high and those with low need for autonomy was determined, and the usability was optimized.

**Conclusions:**

This resulted in a redesigned digital health communication intervention that included message frame–tailoring and had optimal usability. A detailed description of the redesigning process of the PAS program is provided.

**Trial Registration:**

Netherlands Trial Register NL6512 (NRT6700); https://www.trialregister.nl/trial/6512

## Introduction

### Background

In 2018, a total of 22.4% of the adult Dutch population indicated to be occasional smokers [1]. In 2017, an attempt to quit smoking at least once was undertaken by 35.7% of Dutch smokers; however, most smokers did not succeed in stopping smoking permanently [2]. Therefore, it is important to provide effective interventions to support smokers to stop smoking and prevent the relapse of smoking. For instance, computer-tailored interventions appear to be a promising and cost-effective solution [3-6].

Computer tailoring refers to an automated communication strategy intended to reach every individual with tailored messages by adapting these messages to the individual’s unique characteristics and his or her behavioral and motivational state [3,7,8]. Generally, computer tailoring focuses on tailoring the content of health communication interventions [9-11]. An example of a web-based computer-tailored intervention is Personal Advice in Stopping smoking (PAS) [12]. This is a computer-tailored smoking cessation program, which provides tailored feedback based on respondents’ answers to web-based questionnaires based on the Integrated-Change (I-Change) Model [13]. The I-Change Model combines insights from several behavior change theories, such as the Transtheoretical Model [14], Theory of Planned Behavior [15], Social Cognitive Theory [16], and the Health Belief Model [17]. As such, the I-Change Model assumes a person’s behavioral intention to be the most proximal predictor of behavior. In turn, this behavioral intention is proposed to be predicted by three motivational constructs: attitude (represented by perceived advantages and disadvantages of the behavior), perceived social influence (composed of perceived social norms, social modeling, and social pressure), and self-efficacy (a person’s perceived ability to perform the behavior). Furthermore, the I-Change Model includes several premotivational factors: predisposing (eg, past behavioral experiences), awareness (eg, knowledge), and information (eg, message source) factors. Finally, the I-Change Model suggests that although a positive behavioral intention is needed for behavior change to occur, several postmotivational factors play a role in bridging the gap between intention and behavior [18], with perceived barriers to change widening this gap and ability factors (eg, skills and the formation of action plans) narrowing this gap.

Given its theoretical grounding, the PAS program starts with a baseline assessment consisting of questions on sociodemographic characteristics, smoking behavior, and addiction level, but, then, continues to ask questions about the respondent’s intention to quit smoking, attitude, social influence, self-efficacy beliefs, and action and coping planning on how to stop smoking and prevent the relapse of smoking [12]. Then, the PAS participants receive tailored feedback messages based on their answers [12]. Questions and feedback messages alternate, meaning that, participants answer a question—or a set of related questions—and immediately receive the feedback message associated with their answer and, then, receive the next question. At the end of the program, participants receive an overview of their tailored feedback messages, which can be printed and sent to them through email if desired. A detailed description is provided elsewhere [12,19].

An investigation of the effects of PAS among Dutch smokers indicated a significant effect on smoking abstinence, reported 6 weeks after the initiation of participation in the program. However, 6 months after participation initiation, significant intervention effects could no longer be found [19]. In other web-based computer-tailored interventions to promote health behavior, intervention effects also declined after intervention completion [20]. Therefore, and because overall effect sizes remain small [3,20], it is worth exploring additional strategies that might enhance intervention effects. A proposed strategy to improve the effectiveness in addition to tailoring *what* information is presented (ie, content tailoring), is tailoring *how* this information is presented, which is known as message frame–tailoring [10].

Message frame–tailoring refers to an integration of the message frame theory [21] and theory around tailoring of health information [22]. According to the message frame theory, the manner in which information is presented is referred to as framing of information, which contains 2 aspects: selection and salience [21]. Selection refers to choosing several aspects to communicate, and salience is defined as “making a piece of information more noticeable, meaningful, or memorable” [21]. Selecting elements to communicate and making them more (or less) salient to the audience will capture the audience’s attention and, as a result, make the information more likely to be read and processed. This is where tailoring theory comes in, as tailoring of information is also done to increase the likelihood that information is read, processed, remembered, and acted upon. Tailoring is “any combination of information or change strategies intended to reach one specific person and increase the personal relevance of the information for this particular person, based on characteristics that are unique to that person, related to the outcome of interest, and derived from an individual assessment” [8]. Relevance of the presented information for an individual is a key concept in this definition, as personally relevant information may lead to more in-depth information processing [22]. Combining message frame theory with theory around tailoring leads us to the definition of the main concept described in this paper, that is, message frame–tailoring, which is “adjusting the perspective when formulating a message based on people’s individual needs” [10]. In this study, message frames were tailored to respondents’ *need for autonomy*, a theoretical concept derived from self-determination theory (SDT) [23]. Tailoring health messages to respondents’ need for autonomy is important because health messages should not only be read and considered as personally relevant but, above all, should encourage behavior change by increasing the recipients’ autonomous motivation for change. According to SDT, autonomous motivation plays an important role in enhancing the initiation and maintenance of health-related behavior [24]. To be autonomously motivated, people need to experience a sense of volition and freedom to make their own choices. On the other hand, people are likely to not feel autonomously motivated when they feel controlled and experience pressure to think, feel, or behave in a manner that has been imposed on them [25]. In SDT, this prerequisite for autonomous motivation is called fulfilling the need for autonomy [23]. However, according to SDT, individual differences in the need for autonomy exist regarding health and health-related decision-making. For example, some people prefer to choose their own path toward lifestyle improvement, whereas others prefer to be guided by clear-cut expert advice [26,27]. To meet these individual differences in the need for autonomy, the manner of information provision in health messages can be tailored based on this need, to accommodate an individual’s associated processing and communication style preferences and further increase the personal relevance of the message [10].

In our operationalization, message frame–tailoring based on the need for autonomy consists of 2 components: language (ie, autonomy-supportive or controlling) and choice (ie, provision of choice or no choice) [28,29]. Autonomy-supportive language, or noncontrolling language, is defined as “a vocalization that would allow choice or support self-initiation” [30] and can be used for individuals with high need for autonomy. Autonomy-supportive language encourages individuals to take responsibility for their own behavior and make decisions based on their own values [28,30]. This involves minimizing pressure [28] and using words such as *could*, *might*, and *would*. Controlling language is defined as “a vocalization to pressure a person to behave (or think or feel) a particular *way*” [30] and can be used for individuals with low need for autonomy. Controlling language is characterized by the use of commands, orders, imperatives, and suggestive questions [30]. Examples are words such as *must*, *should*, and *ought*. Choice is operationalized as offering participants the option to receive additional information about a certain topic [31]. Tailoring message frames to a person’s need for autonomy may enhance the motivation to change and maintain health-related behavior. As such, message frame–tailoring based on the need for autonomy has been suggested as a promising avenue to explore further [10,26,27,32]. However, to date, to the best of our knowledge, no health-related computer-tailored intervention has been reported that incorporates message frame–tailoring based on the need for autonomy, except the study by Smit et al [10].

### Objective

To investigate whether adding message frame–tailoring based on the need for autonomy increases the effectiveness of content-tailored interventions, the PAS program [12] was redesigned to incorporate message frame–tailoring. This paper describes the process of extending framing theory, tailoring theory, and SDT by redesigning the PAS program to include message frame–tailoring. The redesigning process was conducted in close collaboration with communication science students, experts, and the actual end users (ie, smokers).

## Methods and Results

### Overview

**Figure 1:**
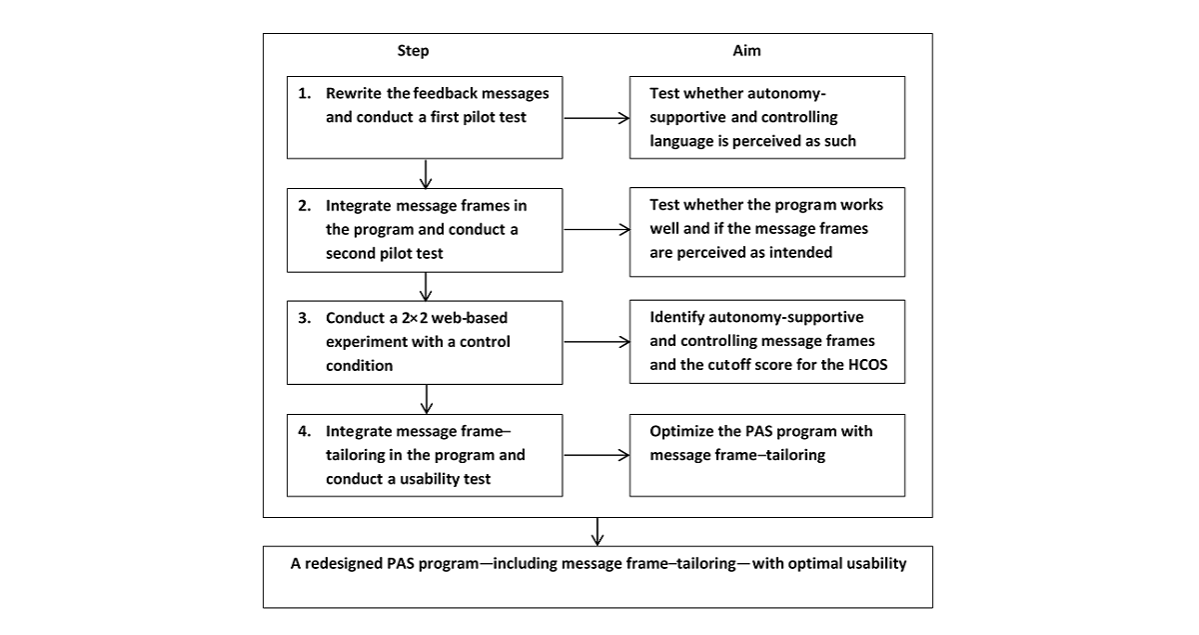
Overview of the redesign process. HCOS: Health Causality Orientations Scale; PAS: Personal Advice in Stopping smoking.

The process of redesigning the original PAS program to incorporate message frame–tailoring consisted of four steps: (1) the feedback messages were rewritten in an autonomy-supportive and a controlling manner and a first pilot test was conducted, (2) the message frames were integrated into the program and a second pilot test was conducted, (3) a web-based experiment with a 2×2 between-participants design with a control condition was conducted, and (4) message frame–tailoring was integrated into the program and a usability test was conducted. An overview of the steps is presented in Figure 1, and the 4 steps are described in detail in subsequent sections. OverNite Software Europe provided technical support and usability advice during the redesign process.

The study was registered in the Netherlands Trial Register (NL6512/NRT-6700).

### Rewrite the Feedback Messages and Conduct a First Pilot Test

To tailor message frames based on the need for autonomy, the message frames needed to be developed. The feedback messages of the original PAS program were rewritten using autonomy-supportive and controlling language strategies. To test whether the language strategies were perceived as such, a pilot test was conducted among communication science students. After consenting to participate, students were randomly assigned to either evaluate the autonomy-supportive or controlling messages. The students received a questionnaire with 5 messages to be reviewed using a 5-point response scale, ranging from very autonomy-supportive to very controlling [33]. Students were also able to write comments on each message in a textbox. A total of 19 students participated (mean age 23.2, SD 1.44 years; 2/19, 11% were men; 15/19, 79% were nonsmokers). Of the 19 students, 10 (53%) students received the autonomy-supportive messages and 8 (42%) students received the controlling messages. Quantitative results were analyzed using an independent sample *t* test (2-tailed) using SPSS (IBM Corp), and the qualitative comments were inspected to identify relevant comments regarding language use.

As the 5-point scale ranged from very autonomy-supportive (rating=1) to very controlling (rating=5), we expected lower scores for the autonomy-supportive messages than the controlling messages. Results showed significantly lower scores for the autonomy-supportive messages (mean 2.46, SD 0.62; *P*<.001) than the controlling messages (mean 3.68, SD 0.32), which means that participants detected the language manipulation as intended. However, based on participants’ comments and inspection of the differences between messages that had high or low scores and those that had average scores, we further emphasized communication style (autonomy-supportive vs controlling). The main change consisted of emphasizing with *signal words* on the type of language style—autonomy-supportive or controlling—used at the beginning of each message (eg, starting the message with “you must...” in a controlling message).

Subsequently, the second message frame element, choice, was added. The following question was included 6 times throughout the program: “Do you want to receive these tips?” which could be answered with “yes” or “no.” In addition, participants were asked if they wanted to choose a quit date themselves. Furthermore, participants could choose whether and for which potential difficult situations they wanted to formulate coping plans to refrain from smoking in these situations.

### Integrate Message Frames in the Program and Conduct a Second Pilot Test

Adding message frames (autonomy-supportive vs controlling language and provision of choice vs no provision of choice) to the feedback messages of the original PAS program resulted in four versions of the program: (1) autonomy-supportive language and no choice, (2) autonomy-supportive language and choice, (3) controlling language and no choice, and (4) controlling language and choice. Besides the 4 message frames, 1 control condition was added to the program, consisting of generic smoking cessation advice with a neutral message frame (ie, the message frame was not manipulated and was the same as in the original smoking cessation intervention) and no content tailoring (ie, the messages were not tailored based on participants’ answers). Following is an example of a control message:

When smokers are stressed, tensed, or dreary, they often find it difficult not to smoke. To help you with this, we give you a few tips. If you find yourself feeling emotional and wanting a cigarette, it’s good to do something else. You can take a walk: the outside air may do you good. Or you can exercise. This helps you to change your mind and reduces the desire for a cigarette.

Examples of feedback messages for the 4 message frames are presented in Table 1. For more examples, refer to the study by Altendorf et al [31].

The next step in the redesign process was conducting a second pilot test to investigate the intervention from experts’ and smokers’ perspectives. Totally, 5 experts (4/5, 80% men) and 11 smokers (8/11, 73% men) participated in the usability test and the subsequent interviews. The experts were working as professionals or researchers in the fields of health communication, public health, eHealth intervention development, and smoking cessation support. First, participants received a link to the website and were instructed to complete the program, while paying attention to the comprehensibility of the program and the feedback messages. Subsequently, a researcher interviewed the participants on the following topics: time taken to conduct the program; comprehensibility of instructions, questions, and answer categories; and awareness of the message frame used. Participants were rewarded with a shopping voucher worth €10 (US $11). During the usability tests and the interviews, the researcher took notes. Results were discussed within the research team to determine whether changes to the program were required to resolve issues mentioned in the feedback.

The participants took between 20-60 minutes (average 35 minutes) to complete the program. As the introduction was perceived as very long, it was shortened. In addition, some questions seemed to be unclear; therefore, a brief instruction was added. Some questions needed to be reformulated (eg, using more familiar words and not using double negatives). In addition, participants wished to receive the summary of feedback messages by email; thus, this option was added. The message frames were generally perceived as intended; therefore, no modifications were made regarding this aspect. After incorporating these changes, the PAS program with message frames was ready for use in the next step.

**Table 1:** Examples of feedback messages for the different message frames.

Condition and process		Autonomy-supportive language	Controlling language
**No choice**			
	Message	You answered that you will succeed in not smoking if you are stressed, tensed, or dreary. You doubt whether you will succeed in not smoking when you are angry. We would like to offer you some tips. If you notice that you are emotional and would like to have a cigarette, you can try to do something else. For example, you could take a walk: the outside air might do you good. Or you could exercise. This might help you to change your mind and it reduces the desire for a cigarette.	You think that you will succeed in not smoking if you are stressed, tensed, or dreary. You doubt whether you will succeed in not smoking when you are angry. You need to do something else when you are emotional and want a cigarette. Take a walk: the outside air often works well. Or go exercise: this should help to change your mind and reduces the desire for a cigarette.
**Choice**			
	Message 1	You answered that you will succeed in not smoking if you are stressed, tensed, or dreary. You doubt whether you will succeed in not smoking when you are angry. Below, you can choose whether you would like to receive some tips on what you can do when you are emotional and would like to have a cigarette.	You think that you will succeed in not smoking if you are stressed, tensed, or dreary. You doubt whether you will succeed in not smoking when you are angry. Below, you can choose whether you would like to receive some tips on what you can do when you are emotional and would like to have a cigarette.
	Choice question^a^	Do you want to receive these tips?	Do you want to receive these tips?
	Answer	Yes	Yes
	Message 2	If you notice that you are emotional and would like to have a cigarette, you can try to do something else. For example, you could take a walk: the outside air might do you good. Or you could exercise. This might help you to change your mind and it reduces the desire for a cigarette.	You need to do something else when you are emotional and want a cigarette. Take a walk: the outside air often works well. Or go exercise: this should help to change your mind and reduces the desire for a cigarette.

^a^If respondents answered “no” to the choice question, the intervention continued without the provision of the tips.

### Conduct a 2×2 Web-Based Experiment With a Control Condition

To tailor message frames based on individuals’ need for autonomy, we had to identify (1) the most autonomy-supportive and the most controlling message frame based on participants’ perceived autonomy support and (2) the cutoff point to distinguish between people with high need for autonomy and those with low need for autonomy. Therefore, a web-based experiment with a 2×2 (language: autonomy-supportive vs controlling; choice vs no choice) between-participants design with a control condition was conducted. An extensive description of the method and results of this experiment is provided elsewhere [31]. However, we summarize the most important outcomes below.

Participants’ need for autonomy was measured using the Health Causality Orientations Scale (HCOS), consisting of 4 vignettes, each of which contained 3 items that could be answered on a 5-point Likert scale (1=low need for autonomy; 5=high need for autonomy) [34]. The primary outcome measure was participants’ perceived autonomy support, which was measured with the Virtual Climate Care Questionnaire, consisting of 15 items that can be answered on a 7-point Likert scale (1=low perceived autonomy support; 7=high perceived autonomy support) [35]. Therefore, the HCOS measured participants’ general need for autonomy and the Virtual Climate Care Questionnaire measured how the participants perceived the messages in the PAS program; that is, whether they perceived them as autonomy-supporting.

Results showed that participants’ perceived autonomy support was generally high (mean 3.46, SD 0.78). On the basis of the results, it was not possible to identify the most autonomy-supportive and the most controlling message frames. Therefore, we decided to follow SDT principles, according to which the provision of choice and the use of autonomy-supportive language enhances an individual’s autonomy [23]. Additional inspection of the data supported this decision, as it appeared from the evaluative comments that especially participants with high need for autonomy mentioned that the word *must* was salient in the advice and not appreciated. Furthermore, especially participants with high need for autonomy chose to receive more information when offered choice and wanted to choose a quit date themselves more often than participants with low need for autonomy.

Regarding participants’ need for autonomy, the results also showed a high overall score (mean 3.87, SD 0.76). The Johnson-Neyman procedure was followed to identify the need for autonomy score at which the three-way interaction of the need for autonomy, language use, and provision of choice on perceived autonomy support changed from insignificant to significant, resulting in a range of 3.8 to 4.4. The qualitative data showed that participants with need for autonomy ≥3.8 more often made unappreciative comments about not being able to set a quit date themselves and about the word *must* used in their feedback messages. On the basis of these results, 3.8 was chosen as the cutoff point of the HCOS to distinguish between individuals with high need for autonomy and those with low need for autonomy.

On the basis of the evaluative comments from participants, some changes were implemented. First, the participants perceived a period of 2 weeks to set a quit date as very soon. In the original PAS program, the quit date was set 4 weeks after participation initiation, but based on results that showed the importance of immediate action when a quit attempt was considered [36,37], it was decided to shorten the period to 2 weeks. To clarify this to the participants, a brief explanation about the benefits of setting a quit date within a short period was added. Participants in the no choice conditions were still given a quit date within 2 weeks, whereas participants in the choice conditions were offered an additional question of whether they wanted to choose a quit date within 2 weeks. On the basis of their answer (“yes,” “no,” or “I am not sure yet”), the participants would see a different calendar to set a quit date (ie, a calendar with dates within a time span of 2 weeks, a calendar with dates with a time span of >2 weeks, or a commenting section to enter a date). Second, participants wished to comment on their former quit attempts. Therefore, a question was added about the number of previous quit attempts, followed by a feedback message in which the participants were asked to note why former quit attempts did not succeed and what they have learned from this. Third, participants wanted to receive more information about smoking cessation support tools (eg, medication or nicotine patches), the costs of smoking, and the use of replacing smoking with other oral stimuli (eg, chewing sunflower seeds). New feedback messages regarding these topics were added. Fourth, it was noted that the program was not suitable for a tablet or mobile phone; therefore, the program was made compatible with different devices. This included automatic adjustments to the screen size, alignment of the buttons (eg, forward and print) and pictures, and centering error messages. Finally, as the participants seemed to have difficulties in answering questions that were presented in matrices, all questions were presented as separate questions.

### Integrate Message Frame–Tailoring in the Program and Conduct a Usability Test

**Figure 2:**
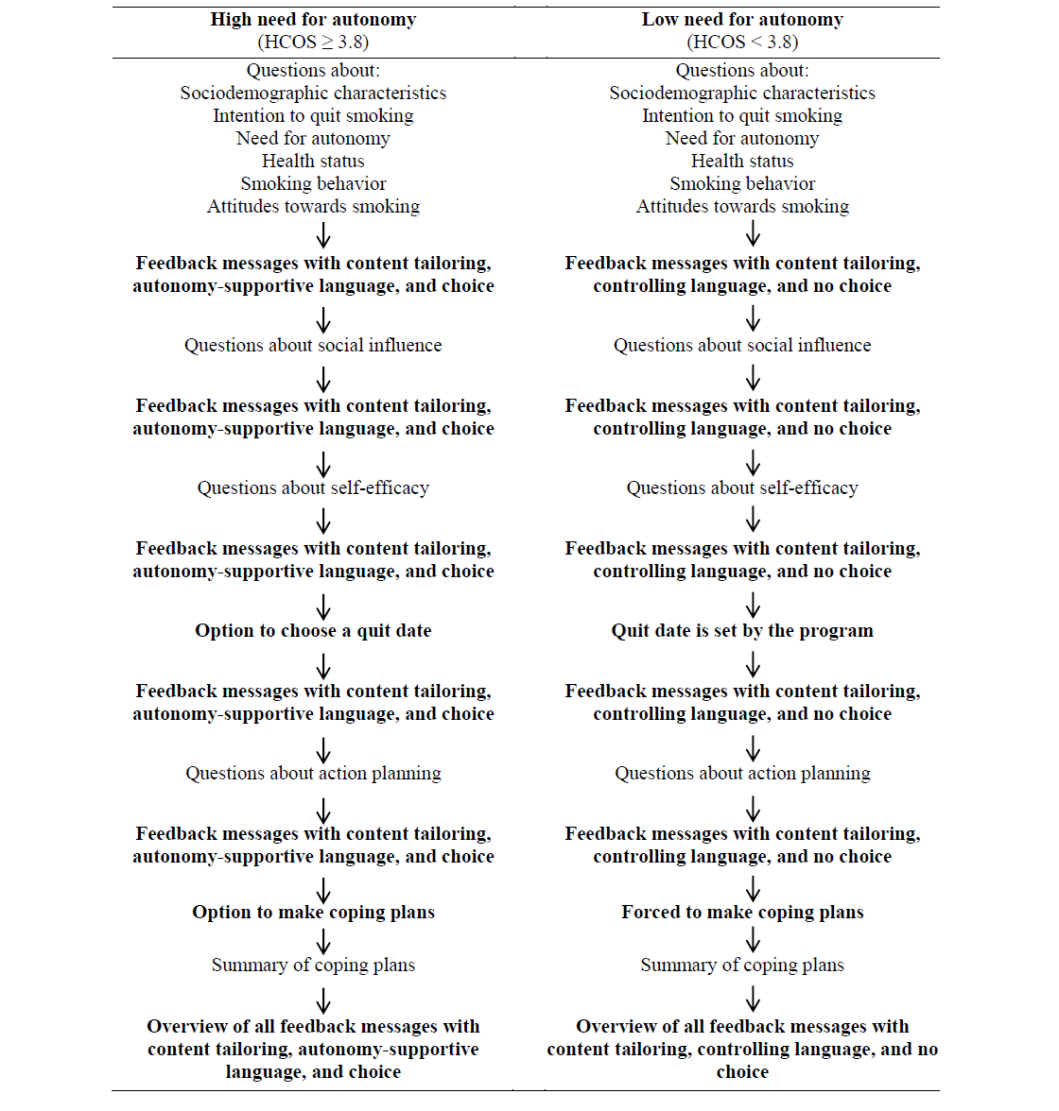
Flowchart of the redesigned Personal Advice in Stopping smoking program that incorporates both content tailoring and message frame–tailoring based on the need for autonomy. HCOS: Health Causality Orientations Scale.

On the basis of the results of the web-based experiment as described in the previous step, the PAS program was developed into a program with message frame–tailoring. The message frame for participants with high need for autonomy (HCOS score ≥3.8) consisted of feedback messages with autonomy-supportive language and choice, and the message frame for participants with low need for autonomy (HCOS score <3.8) consisted of feedback messages with controlling language and no choice. The flow of the redesigned PAS program with content tailoring and message frame–tailoring is presented in Figure 2.

To optimize the PAS program with the tailored message frames, a usability test among experts and smokers was conducted. Regarding user experience, it is argued that, besides the aspect of ease of use, perceived usefulness is also important, which refers to the extent to which the content of the program satisfies the information needs of the participant [38]. Therefore, we aimed to not only identify problems with the use of the program but also to test the content of the program from experts’ and smokers’ perspectives. Experts were working as professionals or researchers in the fields of health communication, public health, eHealth intervention development, and smoking cessation support.

Both smokers and experts were asked to evaluate the program using the think-aloud method, based on which they were asked to verbalize their thoughts while using the program [39]. As verbalizing one’s thoughts while conducting a task is not common, the participants first received a brief task to practice. After completing the usability testing, a semistructured interview was conducted with the smokers to evaluate the program and further explore the problems mentioned. Examples of questions are, “What do you think of the layout of the program?” and “Which are the two most important elements that could be improved?” To identify 80% to 90% of the user issues, 5 to 9 end users were needed [40,41]. The semistructured interviews with experts were based on the heuristic evaluation method [39,42]. The heuristic evaluation method consists of ten heuristic principles, such as simple and natural dialogue, speak the user’s language, consistency, and good error messages [42]. To identify most problems by using the heuristic evaluation method, 3-5 experts were needed [39].

As the program will be used on different devices, smokers and experts were instructed to use the program on a laptop, tablet, or mobile phone. The entire procedure took approximately 1 hour to complete, and the participants were rewarded with a shopping voucher worth €25 (US $27.5). Experts and smokers were recruited through the network of the research team. A total of 5 experts (2/5, 40% were men; 4/5, 80% were nonsmokers) and 7 smokers (mean age 31.86, SD 14.15 years; range 20-62 years; 4/7, 57% were men) participated in the usability test. All sessions were video-recorded, which were selectively transcribed and coded using ATLAS.ti 8.0 [43]. The data were analyzed according to the content analysis approach [44]. Deductive content analysis allows us to go beyond general findings and validate theories and models that guided the research. Inductive content analysis allows for inclusion of codes and categories that are derived from the data in an iterative process [45]. First, codes were derived using the heuristic evaluation method [39,42]. Then, the transcripts were thoroughly read and coded. In the next step, categories were identified and ordered into themes that represented the answers to the research questions. When new themes emerged from the collected data, they were added to the analysis [45]. Initially, 2 transcripts were coded independently by 2 coders (ISvSK and MBA). Disagreements were resolved through discussion, and after reaching consensus, 1 coder continued coding the remaining transcripts (ISvSK).

As we aimed to test the content of the program and identify problems with its use, user experience was divided into 2 dimensions: the content of the program and the usability of the program. Modifications that were made regarding the content of the program included the following: replacing difficult words, shortening the introduction and instructions, reformulating questions, changing the labels of answer categories, and adding the quit date to the summary. Modifications that were made regarding the usability of the program included the following: adding a sidebar to show the different parts of the program, modernizing the logo and the colors, displaying only the core messages in bold font, aligning the illustrations on a mobile phone, adding a red frame signaling an unanswered question on the mobile phone, and adding the option to directly add the quit date to a personal agenda. An overview of the codes, subcodes, definitions, examples, and modifications is presented in Table 2.

It is worth noting that participants also wished to receive feedback messages with more illustrations and that some participants wished for shorter feedback messages with more bullet points. However, as the original feedback messages were effective in supporting smokers to quit smoking [19] and making large modifications to this content would mean retesting the effectiveness of the feedback messages, it was decided to stay as close to the original feedback message format as possible.

**Table 2:** Overview of the codes, subcodes, definitions, examples, and modifications of the usability test.

Code and subcode			Definition		Example		Modifications
**1–Language use**							
	Level and style	Words, concepts, or sentence structures that are unclear or ambiguous. In addition, the use of formal language.		“I don’t even know what this is.” [smoker]		Difficult words were replaced or explained. Spelling mistakes were corrected.	
	Amount of information	The amount of information provided in the program, especially in the instruction, introduction, and informed consent.		“Uhm...well...it’s a lot of text, I know that a lot of low educated people really hate a lot of text.” [expert]		The introduction and instruction were shortened.	
**2–Questions and answers**							
	Number and content of questions	The number, relevance, understandability, and topics of the questions in the program.		“I have to read this sentence four times [...] I find it hard to follow. So, I don’t know what I have to answer.” [expert]		Questions were reformulated.	
	Answer categories	The answer possibilities, way of answering, and unclarity and inconsistencies regarding scale questions.		“Maybe you also need some kind of range here, don’t you? Never, uhm, a few times, very often or I try to do it regularly.” [smoker]		The labels of some answer categories were changed. Some answer options were changed from a textbox to a dropdown menu.	
**3–Feedback messages**							
	Content and number	The number, length, topics, and content of the feedback messages.		“This is good, good arguments here, especially at the end. With time and money, that is good.” [smoker]		A short explanation of the summary of the feedback messages was added. In addition, the quit date was added to the summary.	
	Credibility and relevance	The concreteness, reliability, and credibility of the feedback messages and whether the messages are perceived as true and suitable to support in quitting smoking.		“They are all things that I have already read and heard, but it is all true.” [smoker]		No modifications were made regarding this aspect.	
	Illustrations	The support of illustrations to the text and the number of illustrations.		“I do indeed see here a picture of someone who gives some kind of support, so that does support the text.” [expert]		No modifications were made regarding this aspect.	
**4–Tailoring**							
	Content tailoring	The relevancy of the content for a particular participant.		“I think it’s good that for each answer category a story is told what really applies.” [smoker]		No modifications were made regarding this aspect.	
	Message framing	The use of autonomy-supportive or controlling language and the provision of choice.		“You must, there is a lot ‘must.’ I don’t feel like quitting smoking anymore.” [expert]		No modifications were made regarding this aspect.	
**5–Program structure**							
	Structure	The order and structure of the different parts of the program and whether the order is clear. In addition, the feedback that a participant receives via the progress bar and whether it is possible to return to the program.		“Step 3. [...] I don’t remember so well that I saw step 2, but those steps are a bit out of the blue. Maybe I should be taken a little more by the hand.” [expert]		To make the structure of the program clearer, a sidebar was added, which continuously shows the different parts of the program throughout the completion process, including which part a participant is working on. In addition, the titles of the different parts were made larger.	
	Instructions	The unclarity of content of the instructions, especially concerning how to complete the program and how to answer the questions.		“How to complete the questionnaire. [...] Well, all basic things.” [smoker]		No modifications were made regarding this aspect.	
	Duration	The duration of the program and time required to complete the program.		“It is long, so maybe people will get bored at some point.” [expert]		No modifications were made regarding this aspect.	
**6–Layout**							
	Design	The font size and style, colors, logos, buttons, and illustrations.		“It is very straightforward, so well...there is little...it will be made more beautiful? It is not very inviting.” [smoker]		A modern logo was developed and modern colors were used. In addition, the buttons were changed into modern icons. As the print button seemed to appear too often, this was resolved; some inconsistencies in the font size were also resolved.	
	Readability	The readability of the text, with regard to the layout of the pages, the feedback messages, the summary of the feedback messages, and sentence alignment.		“Shorter sentences, only graphic now in terms of location, you read more easily. This sentence is easier to read than, for example, this sentence, whereby your eye has to go all the way from left to right.” [smoker]		The alignment of the sentences was modified. Furthermore, it seemed unclear which information was displayed in bold font; thus, it was decided to display only the core message in bold font.	
	Layout on various devices	The layout of the program on the various devices (ie, laptop, mobile phone, and tablet).		Someone with a mobile phone: “There is also a picture next to it, that makes the text a bit narrower.” [expert]		Changes were made to the alignment of the illustrations on mobile phones.	
**7–Error messages**							
	Time of appearance	Whether the error messages appear at the right time.		“I have to give an answer, so that works.” [expert]		A red frame signaling an unanswered question was added on mobile phones.	
	Content	The content of the error messages and whether they are perceived as helpful.		“It was clear that it was about the age question. It was not mentioned that it was about the age question, but I think that’s also not possible in an error message.” [expert]		No modifications were made regarding this aspect.	
**8–Features**							
	Calendar	The appearance and ease of use of the calendar.		“You can also place a link here, or something like that so that it will be added to your agenda automatically.” [smoker]		The option to directly add the quit date to a personal agenda was added.	
	Print or email	The option to print or email the summary of the feedback messages and whether this works. In addition, the layout of the printed summary.		“I like that I can print it because it’s a lot to read at once.” [expert]		No modifications were made regarding this aspect.	

**Figure 3:**
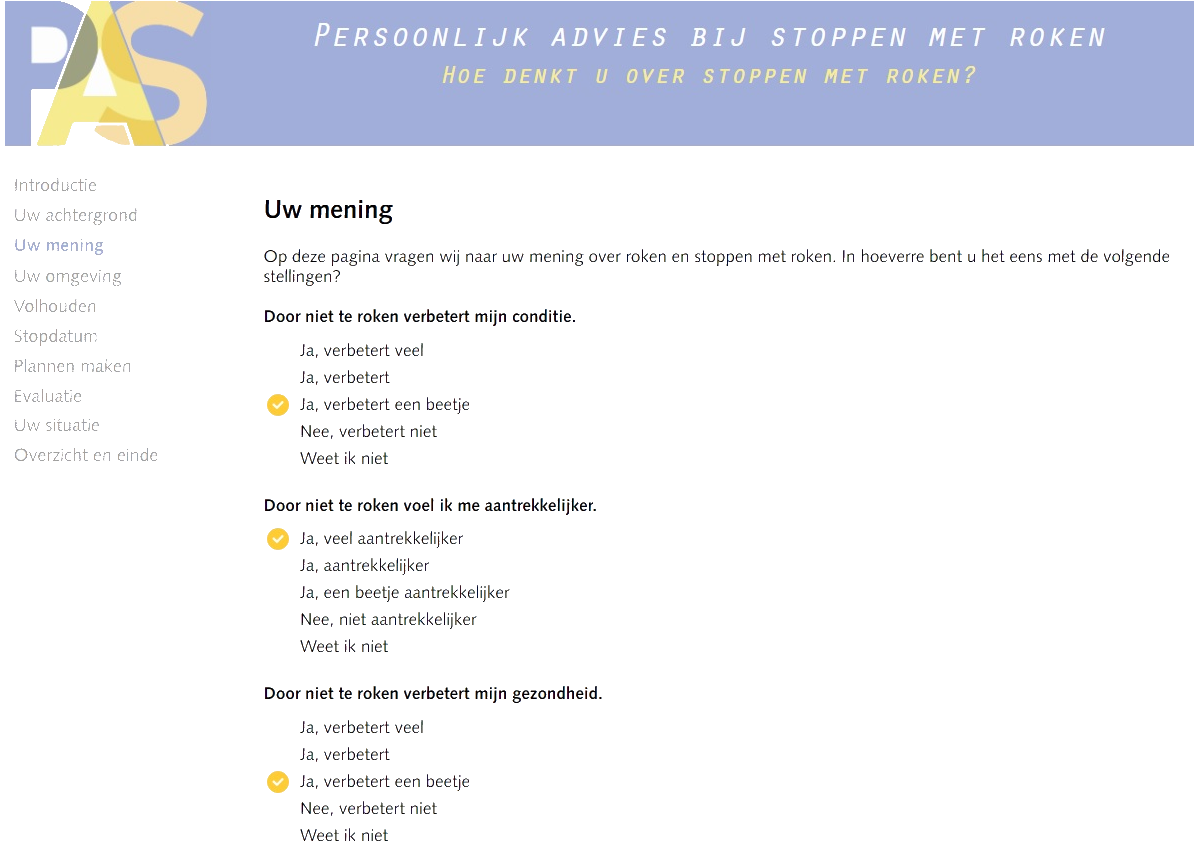
Screenshot that shows the colors, logo, and sidebar of the redesigned Personal Advice in Stopping smoking program.

**Figure 4:**
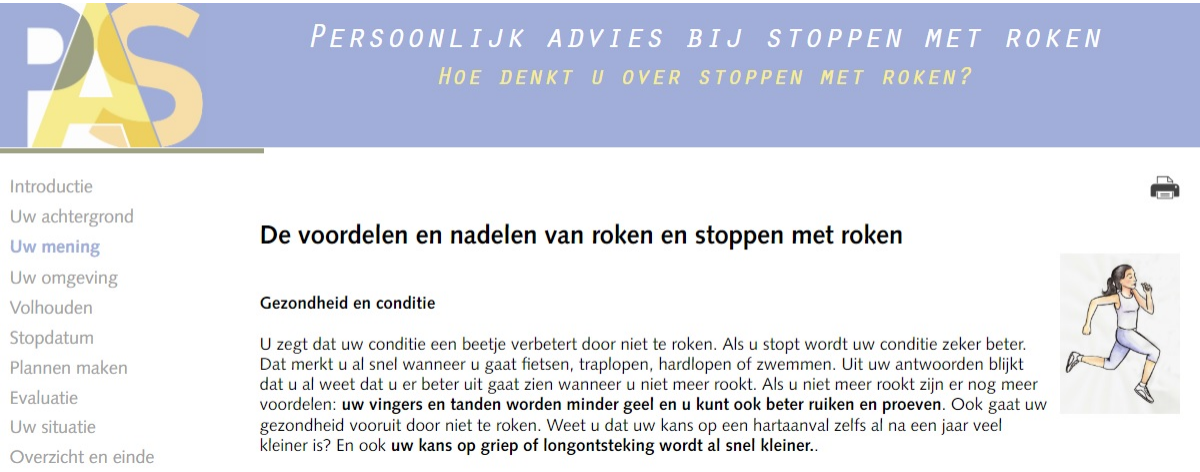
Screenshot that shows the print button and enlarged titles of the redesigned Personal Advice in Stopping smoking program.

After incorporating the modifications in response to the usability test, the redesign process was completed. The redesigned PAS program—including message frame–tailoring—is shown in Figures 3 and 4. For a comparison with the original interface, refer to the study by Smit et al [19]. This redesigned PAS program has been evaluated in a real-life setting for effectiveness and cost-effectiveness; results of the effectiveness evaluation were recently published elsewhere [46], and the results of the cost-effectiveness evaluation are currently being prepared for publication.

### Ethics Approval

This study was approved by the institutional review board of the University of Amsterdam (reference number 2017-PC-7599).

## Discussion

### Principal Findings

This study aimed to extend the message framing theory, tailoring theory, and SDT by including message frame–tailoring in a digital health communication intervention. In addition to tailoring *what* information is presented (ie, content tailoring), we wanted to improve the effectiveness of a digital health communication intervention by tailoring *how* this information is presented (ie, message frame–tailoring). This paper offers a detailed description of the incorporation of message frame–tailoring in a web-based computer-tailored smoking cessation program, PAS. The PAS is taken as an example to explore the effects of—in addition to the known effects of content tailoring—the promising strategy of message frame–tailoring [10]. The process of incorporating message frame–tailoring was conducted in close collaboration with experts and smokers, and a usability test was conducted to optimize the redesigned program. An extensive description of the various methods through which experts’ and smokers’ opinions were included throughout the redesigning process is provided. If the redesigned PAS program is more effective in supporting smokers in their quit attempt than the original PAS program, adding message frame–tailoring to other digital health communication interventions also might be of value.

### Potential Strengths

This study has several potential strengths. First, to the best of our knowledge, this study is the first to explore message frame–tailoring in web-based computer-tailored health communication. The existing literature on message framing mostly includes studies on gain and loss framing, focusing on positive (health) outcomes and costs in terms of health loss, respectively. Regarding tailoring, so far, scholars have mainly tailored the content of web-based interventions; that is, adjusting *what* health information is provided based on individuals’ current health behavior or their self-reported scores on known predictors of the desired health behavior (change) [9]. To build on tailoring theory, advance the strategy of web-based computer tailoring, and further increase its effectiveness, it has been argued that tailoring *how* information is provided, in addition to tailoring *what* information is given, appears to be a promising strategy [10,32]. To our knowledge, message frame–tailoring based on the need for autonomy and operationalized as differences in the provision of choice and the use of autonomy-supportive or controlling language has not been studied previously. By developing a redesigned PAS program including message frame–tailoring, we were able to test whether message frame–tailoring increases the effectiveness of the PAS program. The results of the effect evaluation were published elsewhere [46]. These results indicated that message frame–tailoring based on the need for autonomy can be an effective additional strategy to include in the computer-tailored interventions in addition to content tailoring, but only for people with high need for autonomy and not for people with low need for autonomy. Regarding cost-effectiveness, the combination of message frame–tailoring and content tailoring in web-based smoking cessation programs seems to have high potential for both cost-effectiveness (ie, considering smoking abstinence as the outcome of interest) and cost-utility (ie, considering quality of life as the outcome), thereby providing good value for money. However, when the willingness to pay for each abstinent smoker becomes high (ie, ≥€5000 [≥US $5500]), the addition of message frame–tailoring might not be worth the effort and only content tailoring is preferred. A manuscript reporting on the results of this economic evaluation is currently in the preparation phase. Given these results, it is plausible that adding message frame–tailoring to other web-based computer-tailored or digital health communication interventions will also increase their effectiveness and cost-effectiveness, at least for people with high need for autonomy. This is important because the overall effect sizes of such interventions are generally positive but small [3], limited evidence suggests that such interventions may be cost-effective [4-6], and the internet is highly accessible [47,48]. This paper provides detailed insight into how to integrate this novel tailoring strategy with more traditional tailoring efforts. Furthermore, the application of message frame–tailoring can support the offering of person-centered care by providing feedback in a manner that matches the individual’s preferences and needs, which is one of the key characteristics of person-centered care [49,50]. Moving beyond the tailoring of *what* information is provided to additionally tailoring *how* this information is provided takes into account individual differences in communication style preferences. As such, message frame–tailoring can be considered as the next step in the field of tailored and person-centered health communication. Self-management support interventions, such as the PAS program, have shown to be the most frequent category of interventions with the potential to result in positive health impact for patients with chronic diseases [51]. Therefore, continuation of (research) efforts aimed at the development of improved versions of such programs is warranted.

Another strength concerns the approach taken in developing the redesigned PAS program: scientific and nonscientific experts, smokers, and communication science students were actively involved throughout the process. Involving different types of stakeholders, including end users, in research removes barriers regarding the implementation of research findings and interventions, such as the redesigned PAS program, in practice [52,53]. By describing the process of involving participants in redesigning the PAS program and the valuable insights we have gained as a result, we aim to encourage researchers to involve participants in the development and redesign process of digital health communication interventions.

Finally, we chose to use an existing, already effective intervention [19]. Using an existing intervention as a starting point and adding an additional tailoring strategy to it is a cost-effective and efficient approach because the intervention does not have to be developed from scratch and the time and money that has already been invested are optimally used.

### Potential Limitations

This study also has few limitations. First, in the 2×2 web-based experiment, most participants’ need for autonomy was moderately high. This may not be representative of the need for autonomy in the general population; therefore, the cutoff point of the need for autonomy, based on which the message frame is tailored, might be very high, resulting in suboptimal message frame–tailoring. In addition, research has shown that the need for autonomy might be different for different groups of people [46]. More research is needed to assess the optimum cutoff point for the need for autonomy and to test whether this point differs between subgroups.

Second, we mainly focused on the communication of information in an autonomy-supportive or controlling manner, especially pertaining to the type of words used; for example, *could* in the autonomy-supportive condition and *must* in the controlling condition. Moreover, in the case of the quit date, we also tried to design the way of responding in an autonomy-supportive versus controlling manner; that is, the smokers in the choice conditions received different types of calendars to enter their quit date based on their individual choices in this regard; for example, a calendar to choose a date from or a commenting section in which they could enter the date themselves, whereas smokers in the no-choice conditions were given a prespecified date by the intervention. This method of designing could be further explored as a way to make the interface more compatible with the individual’s need for autonomy. For instance, more questions can be designed such that individuals with high need for autonomy would receive open entry fields, whereas individuals with low need for autonomy would receive limited multiple-choice answers. In addition, how the program is run can be adapted to an individual’s need for autonomy; for example, a fixed order for those with low need for autonomy and a self-determined order for those with high need for autonomy. More attention to the design of the intervention as tailored to an individual’s need for autonomy could possibly further increase the effects of this and similar interventions—therefore, such possibilities warrant more attention in future research.

Third, although using an existing, already effective intervention is a cost-effective and efficient approach, it also has limitations. For example, some participants requested shorter feedback messages with more bullet points or more illustrations. However, as we wanted to be able to make a comparison between the effectiveness of the PAS program with and that without message frame–tailoring, it was decided to make no adaptations regarding these aspects. By changing the content of the program, it would not be possible to attribute any differences in effectiveness between the original PAS program and the redesigned PAS program to the effect of message frame–tailoring. When choosing between developing a new intervention from scratch and redesigning an existing effective intervention, we recommend considering both the cost-effectiveness and efficiency of the approaches and the potential limitations of the approaches, such as (not) being able to meet all participants’ needs regarding the usability of the program.

## Abbreviations

HCOS: Health Causality Orientations Scale
I-Change: Integrated-Change
PAS: Personal Advice in Stopping smoking
SDT: self-determination theory
